# Imperial mission: Jesuits, French diplomacy, and medical education at *l’Aurore* University in Shanghai, 1912–1952

**DOI:** 10.1017/mdh.2024.17

**Published:** 2024-04

**Authors:** Steven Pieragastini, Martin Robert

**Affiliations:** 1 Lecturer, Department of History, Olin-Sang 215, MS 036, Brandeis University, 415 South Street, Waltham, Massachusetts, 02453, United States; 2 Banting Postdoctoral Fellow, Centre for History in Public Health, London School of Hygiene and Tropical Medicine, 15–17 Tavistock Place, London WC1H 9SH, UK

**Keywords:** Shanghai, Aurore, Pasteur Institute, Hanoi, Catholic, Medical Faculty, Birth Control

## Abstract

Between 1903 and 1952, there was a Jesuit and French university in Shanghai called *l’Aurore.* This article focuses on its medical faculty, which operated from 1912 to 1952. It shows that, in a precarious political and military context, *l’Aurore* simultaneously benefited from Jesuit missionary activity and the French quest for imperial influence, without fully identifying with either. The faculty was not an official missionary institution, and most of its hundreds of students were not Christians. However, the Jesuit administration kept a record of baptisms among the students and, based on Catholic principles, encouraged opposition to birth control through courses on ‘medical ethics’ and a special oath that medical graduates had to take. Nor was the medical faculty an overtly imperial institution. It was part of a concession and the result of an alliance between Jesuit missionaries and anti-clerical diplomats of the French Third Republic. Yet, the faculty was key to a French policy of imperial influence designed to compete with other imperial, religious, and private foreign powers active in medical education in China. During the years of war between China and Japan (1937–1945), the faculty consolidated its influence by increasing student numbers and building new infrastructure, whereas its Chinese staff assumed a more prominent role, reinforcing the importance of Chinese medicine in teaching and research. Doctors trained at *l’Aurore* who stayed in China remained active in public health until well into the second half of the twentieth century, even after the medical faculty was abolished by the Communist regime.

## Introduction

Between 1903 and 1952, there was a Jesuit and French university in Shanghai called *l’Aurore*, the French word for dawn, known in Chinese as Zhendan Daxue (Zhendan also meaning dawn in Chinese).^1^ Its students and staff were nicknamed the Aurorians. This article focuses on *l’Aurore*’s medical faculty, which operated from 1912 to 1952. Compared to other medical education institutions in China, this faculty was relatively modest. It was not the only French medical training institution in the country, but it may have been the most influential. For forty years, in a particularly unstable political and military context, *l’Aurore* trained hundreds of medical students, mainly Chinese students, in the French language and under Jesuit supervision. Some Aurorians went on to hold high offices in the Republic of China (1912–1949).^2^

The number of students in all the faculties of *l’Aurore* remained consistently above 500 from the time women were admitted in 1937.^3^ In the last fifteen years of *l’Aurore*’s existence, when enrolment was at its highest, medical students ranged from 26% to over 48% of the total student body, that is, between about 100 and 300 medical students in any one year.^4^ Those who completed their medical studies went on to work throughout China and abroad.^5^ The longevity, scale, and international scope of this medical faculty warrant a closer look, especially because there is a rich historiography on the English-speaking medical institutions in China, but much less on the French-speaking ones, with important exceptions on which this article builds.

We argue that *l’Aurore*’s medical faculty had a significant and lasting impact in China and beyond because it simultaneously benefited from Jesuit missionary activity and the French quest for imperial influence, without fully identifying with either. Through this ‘strategy of ambiguity’, in the words of Yi Ren and Mingzhe Zhu,^6^ the faculty could present itself as a purely scientific institution whose main function was neither religious nor political. It emphasised the usefulness of its professors and medical graduates, particularly in times of armed conflict or epidemics, while catering to the professional aspirations of a certain class of Chinese society.

The first part of this article traces the actions of the Jesuits in the history of the faculty.^7^ The medical faculty had a missionary dimension which it was careful not to advertise in order to avoid criticism. On the one hand, Christians, let alone Catholics, were never a majority at the faculty. There was no religious obligation attached to enrolment. Proselytising was officially extracurricular. On the other hand, the university’s administration kept a record of the religious composition of the student body and Catholic baptisms among Aurorians. It also immersed students in an environment guided by Christian values so that later in life, they would at least not be hostile to the Christian religion and European missionaries in Asia.^8^ At the medical faculty, teaching more directly bore the mark of Jesuit proselytism in the form of mandatory courses on ‘medical ethics’, which condemned the growing birth control movement from the 1920s onwards. This extended to the obligation for medical graduates to take an oath in which they pledged to resist birth control as doctors.

The second part argues that *l’Aurore*’s medical faculty was a key element of French imperial policy in Asia. It operated in the French Concession of Shanghai (1849–1943), a loosely controlled quasi-colony. Its funding came mainly from French diplomatic subsidies and Jesuit land holdings to give it a competitive edge over other imperial powers operating medical institutions in China, including Britain, the United States and Germany, as well as Protestant missionary organisations and private foundations, most notably the Rockefeller Foundation. The majority of the students at *l’Aurore*’s medical faculty were Chinese, although there was significant international diversity in the student body, extending the faculty’s reach beyond China. Staff consisted of doctors from France, Chinese doctors who had been trained by the French in Shanghai, some doctors from other nationalities and a few Jesuits. The fact that the medical degrees awarded by the faculty were recognised outside China enabled many Aurorians to continue their medical training or work abroad, particularly in Europe and North America, forming associations of Aurorians. This strengthened French and Catholic networks internationally, just as the French imperial presence was at its geographical and demographic peak.^9^ It also set this faculty apart from the medical schools in empires that offered only basic training without entitling doctors to practise outside a strictly defined colonial territory.

The third and final part examines the last years of the faculty’s existence, marked by the outbreak of the Second Sino-Japanese War (1937–1945) and the abolition of *l’Aurore* as a university under French and Catholic administration in 1952. During the war, the faculty increased its enrolment and built new premises. With the departure of many French students and staff for military service, the influence of Chinese faculty members in medical education and health care grew. This facilitated experiments combining what was increasingly called traditional Chinese medicine with the bacteriological methods of the Pasteur Institute founded in 1938 in Shanghai. The influence of *l’Aurore*’s medical faculty lasted after the Communist Party came to power in 1949. Some Aurorian doctors continued to work in China to meet the country’s urgent medical needs despite criticism of the religious and imperialist framework of their training. Many Aurorians maintained contact with each other, and an association of alumni was later formed.

In addition to the historiography of medicine in Republican China and of *l’Aurore* University, various sources, mainly in French and English, but also some in Chinese, are used, particularly the archives of the French empire in Aix-en-Provence (the *Archives nationales d’Outre-Mer*), the *Bulletin* (1919–1949) published by *l’Aurore*’s medical faculty, and the archives of the French Jesuits in Vanves (France).^10^

## Creating a Jesuit university in Shanghai

The project that led to the creation of *l’Aurore* University began to take shape in the early 1870s when the Chinese Jesuit Ma Xiangbo (1840–1939) sought to establish an educational institution that would combine the study of classical Chinese texts and culture with a Western-style liberal arts education.^11^ He was the headmaster of the elite Jesuit *Saint-Ignace* College in Shanghai’s Xujiahui district. In 1876, due to disagreements with the French Jesuits who ran the Kiang-nan (Jiangnan) mission – for the region south of the Yangtze River where Shanghai is located – over the educational use of classical Chinese texts, Ma left *Saint-Ignace* College and the Jesuits. However, he maintained links with the order and a desire to see his bicultural, syncretic curriculum realised. After working for Li Hongzhang (1823–1901), a very prominent political leader in the late Qing period, and travelling extensively, Ma returned to Shanghai around 1898. He reconciled with the Jesuits and ultimately donated most of his property to them to build an elite academy in keeping with the idea he had been nurturing for more than two decades.^12^

The Jesuits supported Ma’s project, and the French ambassador was contacted about it. During a strike, some of Ma’s students and colleagues at Nanyang Polytechnic College, where he was then teaching, encouraged him to set up an institution of higher education, for which Ma provided land and some of the initial funding. The inauguration took place in early 1903, and *l’Aurore* (dawn) was chosen as the institution’s name to express a desire for renewal driven by a spirit of mutual enrichment between Europe and China.^13^ However, in 1905, Ma and the Jesuits again locked horns over various aspects of administration and curriculum. Ma led a group of teachers and students to found a new institution, Fudan University, whose name could be translated as ‘revived Zhendan’, an implicit criticism of the Jesuits.^14^

After Ma’s departure, *l’Aurore* was re-established with a French-style curriculum and moved in 1908 from Xujiahui to Lujiawan, an area that later became part of the French Concession of Shanghai. Its longest-held address was on Dubail Avenue.^15^ Admission to *l’Aurore* required a good command of French, the language of instruction, and therefore French secondary schools in China became one of the main pools for recruiting students.^16^ It was also possible to gain admission to the university by completing the in-house preparatory course.^17^

While *l’Aurore* was founded in 1903, its medical faculty was not created until 1912. Physics, chemistry, and science courses became pre-medical courses in 1909. Two French doctors were recruited to teach medical courses from 1912 and take the students to the nearby Catholic *Sainte-Marie* Hospital for clinical training.^18^ As it grew to treat thousands of patients (5869 hospitalised in 1933–1934, 68.2% of them were Chinese), this hospital became instrumental in the development of the medical faculty.^19^ Also nearby was the French *Saint-Antoine* Hospital, which served the poorest inhabitants of the area and was involved in the teaching of medicine at *l’Aurore.* ^20^

Through what Jean-Paul Wiest, and more recently Yi Ren and Mingzhe Zhu, have aptly described as ‘indirect evangelisation’, *l’Aurore* was designed to selectively recruit students from well-placed families to instil a Christian ethos in the Chinese elite, without explicitly serving as a means of conversion to Catholicism.^21^ Despite its Jesuit administration, *l’Aurore* was open to students of any religion or none. However, the Jesuits kept statistics on the religious affiliation of their students, celebrating in particular Catholic conversions. Between 1919 and 1946, there was an average of about ten baptisms annually among students across the university, for a total of 274 baptisms in twenty-eight years.^22^ This figure does not include students who were already Christians before enrolling. According to partial data from the 1940s, Catholics and catechumens together accounted for between 29% and 38% of all students at the university. The proportion of Catholics was higher among those who graduated, ranging from 38% to 58% over the same years.^23^ The number of Orthodox, Jewish, Protestant, and Muslim students was also recorded, but it was the category of ‘pagan’ that was consistently and by far the largest, exceeding all Christian denominations combined.

Although not a majority, Catholic students received special support from the administration, notably through the campus church and associations such as the St. Luc Association for Catholic doctors.^24^ Graduates also had access to Catholic missionary networks throughout China. The *Bulletin* of the medical faculty regularly published advertisements targeting Aurorians seeking to work in Catholic hospitals in various parts of the country.^25^ These medical positions seem to have been open to all Aurorian doctors, Catholic or not. Thus, *l’Aurore* became a major supplier of general practitioners and staff for Catholic medical institutions across China. Some Aurorians even went so far as to join Catholic orders, including Alice Pan Ing-cheng, who abandoned her medical studies after six years to become a Carmelite nun in Shanghai.^26^

Religious openness and restraint in Catholic proselytism broadened the university’s recruitment base. It was also a way of not provoking anti-clerical sentiment among elements of the French Foreign Ministry, from which *l’Aurore* received much of its funding. The French Third Republic (1870–1940) was going through a particularly anti-clerical period when the university was founded.^27^ Nevertheless, the French government was not averse to using the infrastructure and resources of well-established Catholic missions for colonial expansion when it seemed the best option available.

There was, however, one aspect of teaching at *l’Aurore* in which the Jesuits asserted their views more openly. It was the course on ‘medical ethics’, particularly when it came to birth control. The professor of ‘medical ethics’ from 1919 was the Jesuit Father Georges Payen, who taught moral theology at the French Seminary in Shanghai for thirty-five years.^28^ The faculty’s focus on ‘medical ethics’ was based on Payen’s lengthy 1922 textbook titled *Déontologie médicale d’après le droit naturel: devoirs d’état et droits de tout médecin* (‘Medical Ethics According to Natural Law: the Duties and Rights of All Doctors’). The target audience was Aurorians, but the textbook was widely distributed in China and beyond, with two new editions in French (1928, 1935) as well as translations at least into Spanish and later Chinese.^29^

Payen’s teaching and writings were mainly aimed at medical students and encouraged opposition to birth control. The birth control campaign was taking shape around the world and emerged in China when its most visible American advocate, Margaret Sanger, founder of the American Birth Control League, visited Beijing and Shanghai in April 1922.^30^ Sanger’s case for birth control was based on arguments linked to eugenics and what was known as ‘neo-Malthusianism’, whereby a selective approach to reproduction was seen as beneficial to society as a whole. This, according to Mirela Davis, ‘gave the birth control movement an aura of future-oriented progressivism’.^31^ Public debates about overpopulation were intensifying around the world, particularly in medicine, and the notion of eugenics was being introduced and discussed in the Chinese scientific sphere, as elsewhere.^32^ In France, reflecting the growing movement, the French Eugenics Society (*Société française d’eugénique*) was founded in 1913.^33^ ‘Although brief’, writes Michelle T. King, ‘Sanger’s visit launched a flurry of coverage in Chinese newspapers and periodicals, introducing urban Chinese audiences to neo-Malthusian theories on the importance of limiting population growth according to eugenic principles, as well as disseminating contraceptive information’.^34^ From that point on, the teaching of medical ethics at *l’Aurore* became increasingly focused on Catholic opposition to birth control.

Around 1936, Margaret Sanger was expected to visit China again. Although this second trip was eventually cancelled, *l’Aurore*’s medical faculty prepared a special issue of its *Bulletin* devoted entirely to denouncing birth control, to be published in time for Sanger’s planned visit.^35^ All the articles in the issue were written by Jesuits: two in French and the other three in Chinese, English, and German, respectively.^36^ Just the previous year, in 1935, a revised edition of Payen’s textbook on medical ethics had been published. In the new chapters, Payen condemned euthanasia and what he termed ‘false eugenics’, recommended medical examinations before marriage but dismissed the idea of a state-guaranteed premarital certificate and strongly opposed sterilisation except for vital therapeutic reasons. François Veuillot was one of the leading French Catholics to praise the new edition.^37^ The main principle Payen sought to defend was that the Christian religion alone provided a moral framework that made it possible to uphold the sanctity of life. Consequently, he argued that the state could only intervene in matters of procreation in accordance with natural law, which he saw as rooted in religion.

This led Payen to believe that no birth should be prevented once a child had been conceived if it was viable for the mother and the foetus, regardless of whether it would result in a child known by the doctor to be disabled.^38^ Citing doctors in the United States, Switzerland, and England, Payen worried about the persuasiveness of eugenic arguments in his time. This edition appeared just after Adolf Hitler came to power, prompting Payen to mention in a footnote on sterilisation: ‘we know what is happening in Germany as we write’.^39^ In arguing that eugenic sterilisation was immoral and unscientific (distinguishing it from therapeutic, punitive, or voluntary sterilisation), Payen cited the papal encyclical *Casti Connubii* of 31 December 1930, in which Pope Pius XI condemned various aspects of eugenics and reaffirmed Catholic doctrine on the family. Payen’s moral outlook was thus shaped by Catholic morality, yet it was also informed by his contact with clinical practice at *l’Aurore.* He argued, for example, that abortion could be morally justified in certain cases, such as when the uterus was affected by a tumour during pregnancy or during an ectopic pregnancy, when surgery was the only way to save the mother’s life, provided that the primary purpose of the procedure was not to remove the foetus.^40^

The medical students at *l’Aurore*, whether Catholic or not, had to make an active commitment to these ideas. When they graduated, *l’Aurore* required them to take an oath specific to the institution, which included this passage: ‘I will use all my influence to combat the theories and practices of birth control. Life being a sacred thing, I will treat it with a sovereign respect in all my patients. In particular, I will take care to save the life of the mother as well as the child.’^41^ Many medical graduates of *l’Aurore* went on to become public critics of any form of birth control.^42^ An early prominent graduate was Song Kouo-ping (Guobin in pinyin, 1893–1956). Aside from translating Payen’s textbook into Chinese, Song published a number of works on medical ethics as well as works on the translation of medical terminology.^43^ He was among the most prominent physicians in Shanghai in the 1930s and a staunch opponent of abortion and birth control, which he associated with the practice of infanticide that was widespread among the rural poor but somewhat exaggerated by foreign missionaries and inaccurately determined to be a uniquely Chinese phenomenon.^44^ Song was effective at drawing on Confucian texts to link ‘infanticide’ (i.e. birth control) to the discourse of ‘national weakness’ that was pervasive among Chinese intellectuals at the time. Therefore, Aurorians were able to have an outsize influence on the discourse of medical ethics in China.^45^

In short, *l’Aurore* was composed of and dependent on a majority of students and funders who were not Catholics, and it did not present itself as a Catholic missionary institution. However, in their administrative records, the Jesuits running the institution took an active interest in its ability to convert students, especially Chinese students, to Catholicism. The main exception to this missionary discretion was the ‘medical ethics’ courses, particularly in their condemnation of birth control about which the Jesuits took the liberty of expressing assertive views that had to be embraced by the students.

## French imperial strategies

Although part of a university founded and run by Jesuits, the medical faculty of *l’Aurore* could not have grown to the size it did without the support of the French state and its diplomacy, which at the time was very anti-clerical. The French Third Republic (1870–1940) was pursuing a policy of imperial expansion in Asia, with French Indochina, in what is now Vietnam, Laos, and Cambodia, as its main base. This was particularly true under Paul Doumer’s governorship of Indochina (1897–1902). A left-wing politician, Freemason and future President of the French Republic, Doumer sought to consolidate the French colonial administration by means other than military force, notably through infrastructure projects such as railways but also through educational institutions, including medical schools.^46^ This policy extended particularly towards southern China, which had become a French zone of influence by the turn of the twentieth century.^47^

Symbolising France’s imperial ambitions in China, the capital of Indochina was moved from Saigon to Hanoi, closer to the Chinese border, in 1902.^48^ In that year, a French medical school was established in Hanoi in collaboration with the leading French physician Paul Brouardel.^49^ From the outset, the French administration attached both geopolitical and medical importance to the creation of this medical school in Hanoi. Providing medical education and health care was seen as a way of winning the sympathy of the local population in the belief that ‘medical training […] would be as effective as the apparatus of our military force in establishing our domination on the banks of the Mekong and the Red River’^50^. The Hanoi school aimed to train ‘health officers’ rather than doctors, as Laurence Monnais has shown.^51^ In other words, the idea was not to create a school like those that existed in metropolitan France but rather one that would provide local students with basic medical training to meet the needs of the colony. The medical school in Hanoi was explicitly modelled on the French medical school in Pondicherry, the main French trading post in India.^52^ It was also modelled on some of the British medical schools in India, which trained practitioners in basic techniques to provide care in small Indian towns.^53^

From 1907, the French medical school in Hanoi planned to admit Chinese students who would then return to China to practise.^54^ As Florence Bretelle-Establet has analysed, medical training of Chinese students by French doctors also began during this period in the French zone of influence in southern China, including in Kunming and Guangzhou.^55^ Meanwhile, French medical education was introduced in Tianjin in northeast China.^56^ It was in the context of the development of French medical education as a means of imperial influence that the project of a French medical faculty in Shanghai took shape.

The career of the French doctor Jean-Augustin Bussière (1872–1958) was the embodiment of this French policy of influence through medicine. Bussière graduated from the *École de médecine de la Marine et des Colonies* in Bordeaux. In 1900, he became head of the health service for French colonial establishments in India and director of the Pondicherry medical school. A follower of Pasteur who had worked in Senegal, Bussière was also sent to Saigon (1901–1902) and Tehran (1903–1909).^57^ As early as 1902, he had contacted the French political authorities to propose the creation of a hospital to treat Chinese patients in Shanghai, but this proposal was rejected for lack of funds.^58^ In 1913, Bussière moved with his family to Beijing,where he lived for most of his life. He played a key role in the development of *l’Aurore*’s medical faculty in Shanghai, quickly becoming its dean and remaining honorary dean until at least 1948.^59^ This made him one of the few non-Jesuits on the administrative staff of *l’Aurore*, at least in the pre-war period.^60^

In supporting *l’Aurore*’s medical faculty, the French authorities were particularly keen to compete with other imperial powers in China such as the British, Germans, and Americans, whose influence was partly due to their medical schools.^61^ According to Pierre Huard, twenty-six European-style medical schools existed in China in 1916 with around 1,940 students. Thirteen of these had been established by missionary organisations, and many had grown out of courses first given in hospitals.^62^ The hefty indemnity imposed on the Qing dynasty after the Boxer Rebellion (1899–1901) funded many medical institutions with American or European connections.^63^

In practice, *l’Aurore* participated in the creation of an imperial medical market in Asia. Protestant missionaries and the Rockefeller Foundation played a key role in this market, particularly through the China Medical Board established in 1914.^64^ In 1933, the vice-president of the Rockefeller Foundation, Selskar Gunn, announced plans to build a medical school on land adjacent to *l’Aurore*, a project that the Jesuits were only able to thwart by organising the purchase of the land.^65^ It was difficult for the French to compete with such rivals. But this burgeoning medical market was also a source of income for *l’Aurore*, whose medical *Bulletin* published advertisements for medicines and medical equipment from German, British, American, and French companies with offices in Shanghai.^66^

Japan’s influence in the Chinese medicine world was becoming no less significant.^67^ In addition, China was developing new antagonisms of its own when it came to medicine. Chinese medicine was then in the process of being redefined in the face of foreign forces, and by the end of the nineteenth century, it was institutionalised in a handful of medical schools before becoming more widespread in medical education from the early twentieth century.^68^ Around the same time, as Bridie Andrews explains, there was a push by Chinese nationalists to limit the influence of traditional Chinese medicine, which they saw as a hindrance to China’s modernisation.^69^

French diplomats were thus looking to increase their influence in China’s already crowded medical market. After the failure of attempts to establish a secular French medical school in Shanghai, the numerous and well-organised medical, educational, and charitable institutions of the Catholic missions seemed the best means of realising French ambitions for influence in Asia, despite the French government’s anti-clerical tendencies.^70^ From 1913 onwards, therefore, *l’Aurore* and its medical faculty were largely funded by the French Foreign Ministry and, shortly afterwards, by the municipality of the French Concession in Shanghai. The condition was that *l’Aurore* promoted itself as a French institution and that its curriculum and staff served French interests – which it did.^71^

Comparatively modest and facing stiff competition, *l’Aurore*’s medical faculty tapped into the desire of some Chinese to gain access to the French medical world at a time when the name of Louis Pasteur, for one, had an international resonance. French diplomacy enabled *l’Aurore* to recruit French professors of medicine.^72^ Several professors at the medical faculty had studied in Bordeaux, perhaps due to the presence of a colonial medical school there.^73^ In addition to the French professors, a number of Chinese individuals were mentioned as having contributed to the establishment of the medical faculty.^74^ In 1917, Zhu Zengzong and Wang Zhenshi became the first to graduate in medicine at *l’Aurore.* ^75^

Anne Frédérique Glaise estimates that there were a total of eighty-nine medical graduates at *l’Aurore* between 1917 and 1934. By 1938, the number had risen to 154.^76^ This was only a fraction of the number who enrolled, contributing to the university’s reputation for rigour and selectivity. In medicine, part of this reputation was based on the fact that, unlike many colonial medical schools, *l’Aurore*’s medical faculty did not aim to train subordinate medical personnel. Instead, it was designed to train full-fledged doctors who could travel and expect to work as doctors in other countries, including France, albeit perhaps with complementary training.^77^ The university’s preparatory course was considered equal to the French *baccalauréat.* Aurorians could therefore be exempted from preparatory courses if they continued their medical studies in France.^78^

The structure and content of the medical programme at *l’Aurore* soon became essentially identical to those in France.^79^ The emphasis on learning anatomy through dissection certainly helped to legitimise the curriculum in the eyes of the French medical authorities. Within two years of the faculty’s establishment, human dissection was officially authorised in China.^80^ By 1935, the University *Bulletin* could boast of the ‘large number of “subjects” the faculty is able to procure’.^81^ The ‘Sacred Heart Hospital’, the ‘Franciscan Hospitals’, and the ‘Charitable Society’ in Shanghai were mentioned as sources of bodies for dissection, showing the interaction between the faculty and Shanghai’s Catholic and charitable organisations.^82^ If there was one thing Shanghai had in abundance at the time, it was abandoned bodies. In the French Concession alone, an organisation dedicated to collecting them recorded no fewer than 73,698 abandoned human corpses between 1929 and 1938.^83^ There seems to be no direct evidence of a link between them and dissection by medical students, but the fact is that Shanghai became known as a place where it was easy to dissect a human body during medical studies (unlike Taiwan, for example), which was essential to gain recognition in French medicine.^84^

*L’Aurore*’s influence in China and abroad grew as it admitted more students and built new infrastructure, including the creation of a school of dentistry in October 1933.^85^ A nursing school was added in 1934.^86^ From 1937, women were admitted to study at *l’Aurore*, including medicine, although it is not clear to what extent their education was separate from that of male students. In medicine, the number of female students remained relatively small: eight in 1940 and fifteen in 1941.^87^ Marriages between Aurorians, notably within the same cohort, were not uncommon.^88^

From the outset, *l’Aurore* and its medical faculty were of international stature. *L’Aurore*’s medical programmes, tailored to French expectations, allowed it to develop close ties with Paris, Lyon, and other French cities, as well as with Catholic universities in Belgium and the French medical school in Beyrouth.^89^ At least thirteen Aurorians studied medicine in Paris. Six Aurorians studied in Louvain, three in Brussels and at least one each in London, Edinburgh, and Marseille. Chinese Aurorian Ou Kouang-yin was awarded a silver medal by the Paris Faculty of Medicine for his thesis completed in Paris.^90^ Some returned to take up teaching posts at *l’Aurore*, such as Dr Song Kouo-ping (Guobin), mentioned above, who graduated in medicine from *l’Aurore* in 1921 and then studied in Paris on a scholarship, including at the Pasteur Institute, before becoming professor of bacteriology at *l’Aurore.* ^91^ A small minority of Aurorian doctors went on to work in other countries, while usually maintaining close links with *l’Aurore.*

While most of the students and an increasing proportion of the professors were Chinese, the student body included several other nationalities, mostly from Europe and Asia but also from regions as far afield as South America and the Middle East.^92^ The largest national minority at *l’Aurore*, even surpassing the French, were the Russians.^93^ The medical faculty’s library reflected this internationalism, offering the latest issues of medical periodicals in English, French, Spanish, Portuguese, and Dutch, and regularly receiving book donations from personal libraries or medical organisations from different countries.^94^ The same internationalism was evident in the contributions to the medical faculty’s *Bulletin* and in the scientific events and medical associations in which the Aurorians participated in Nanjing, Hanoi, or Strasbourg or that they hosted in Shanghai.^95^

Aurorians generally kept in touch with each other. This strengthened international Francophone and Catholic networks at a time when the French imperial presence was growing worldwide. Several alumni associations were formed, and an alumni newsletter was published in the early 1940s.^96^ From 1941, the faculty held a monthly radio broadcast to provide news of its activities.^97^ In the second half of the 1940s, the Jesuit and former missionary to China, Jean de la Largère (1905–1973), organised monthly meetings for Aurorians living in Paris.^98^

To sum up, the French state, despite its anti-clericalism, largely financed *l’Aurore* to serve its imperial policy in Asia and beyond. Specifically, *l’Aurore*’s medical faculty competed with medical institutions run by other foreign powers in China, whether imperial, religious, or private, in a context where the Chinese government and those who were increasingly described as traditional practitioners also provided medical education. In this competitive medical education market, *l’Aurore* offered a medical curriculum equivalent to that available in France. Aurorians could look forward to rewarding careers with access to international Catholic and French-speaking networks. In turn, the university’s high-profile alumni were likely to support their alma mater.^99^ Some Aurorians, particularly the Chinese, went abroad to study or work, whereas a significant proportion of students from other countries came to *l’Aurore*, strengthening the faculty’s international character. Schools of nursing and dentistry, as well as associations of Aurorians in China and overseas, increased *l’Aurore*’s influence in medicine.

## Lasting impact on the medical world

With nationalism and opposition to imperialism on the rise in China, it was not obvious that the Chinese political authorities, of any persuasion, would allow *l’Aurore* to operate.^100^ Nevertheless, *l’Aurore*’s medical faculty kept growing. Like its rivals, it could thrive because of a rapidly expanding public health environment in which it sought to become indispensable.^101^ To maintain recognition by the Chinese authorities, the Jesuits focused on scientific education, avoided open proselytism, and tried to remain independent of both French and Chinese politics, while appointing Chinese members who were sympathetic to their cause to the university’s board of directors.^102^ However, *l’Aurore* was not insulated from politics and periodically faced criticism from the students, particularly for accommodating changing political regimes and discouraging political dissent and engagement.^103^

For the medical faculty in particular, the most effective way to navigate the profound political upheavals of the time, marked by hostilities between Republican and Communist forces, was to emphasise the usefulness of medical work especially in wartime. Sometimes the Aurorians travelled to where the fighting was taking place, such as the battles between warlord troops around Bengbu in Anhui Province in 1926, where they tended to the wounded.^104^ But war could also come to them, as it did during the Sino-Japanese fighting in early 1932, when the Catholics’ relatively safe and neutral position enabled them to organise aid for soldiers and civilians.^105^

When the major battle of the Second Sino-Japanese War (1937–1945) began in Shanghai in 1937, Aurorian medical and nursing students once again volunteered to treat the wounded.^106^ This conflict led to a refugee crisis even worse than that of 1932.^107^ By April 1938, Aurorians had treated hundreds of wounded soldiers and around 50,000 refugees, leading the *Sainte-Marie* Hospital to open up new wings and earning it the silver medal of the French Concession.^108^ The refugee crisis exacerbated long-standing difficulties in preventing infectious diseases, causing the French Concession to rely even more heavily on civil society and international organisations.^109^

These were difficult times for *l’Aurore* and indeed for China, especially during the Japanese occupation of the Shanghai Concessions from December 1941 to 1945. Daily life was made particularly difficult by unreliable rice supplies and inflation.^110^ In 1943, the French Concession was dissolved by the Japanese occupying forces in order to strengthen the legitimacy of Wang Jingwei’s government, which collaborated with the Japanese Empire.^111^ The most significant interruption to *l’Aurore*’s activities during the war occurred between March and August 1945 when the Japanese Army requisitioned the university buildings, leading to a massive relocation of the institution until the end of the war.^112^

Nonetheless, this was also a time when *l’Aurore*’s medical, nursing, and dental sections (the latter forced to close between the outbreak of the war and 1940) tended to grow in student enrolment and prestige.^113^ The medical faculty reported 145 students in 1939, 175 in 1940, and 196 in 1941, claiming to have awarded fifteen degrees in 1940, twenty-five in 1941 and ten in 1943. The number of nursing graduates also increased.^114^

This growth coincided with major changes in the faculty staff. Contact with Europe became more difficult during the war, and many of the French professors and students were called up to serve in France.^115^ France was defeated and occupied by Germany between 1940 and 1944. Reports suggest that the vast majority of the 2,497 French residents in Shanghai’s French Concession in 1942 supported (not necessarily openly) Charles de Gaulle and the Resistance against Germany.^116^ Indirect evidence indicates that Nazism and the fate of France under the Vichy regime were of serious concern to at least some members of the faculty.^117^

The case of Dr Edith Mankiewicz (née Edith Marion Meyer) illustrates how *l’Aurore* could even become a place of refuge for doctors fleeing Nazism. Born in Leipzig in 1910, Mankiewicz fled Germany with her husband, the lawyer Harald Mankiewicz (1905–1993) after the Nazis came to power. Both were in danger because of their Jewish ancestry. They settled in Lyon, France and became French citizens in 1938. The following year they converted to Catholicism.^118^ When France fell into the hands of the German army, Edith Mankiewicz’s medical studies enabled her to become professor of bacteriology at *l’Aurore.* She was one of the few female professors at the medical faculty, if not the only one. Mankiewicz moved with her husband to Shanghai in 1941 and they both worked at *l’Aurore* as professors until 1945.^119^ They then moved to Montreal, Canada, where Edith Mankiewicz spent the rest of her life as a doctor and professor of medicine. Stripped of her French nationality by the Vichy regime in 1944, she was decorated by General de Gaulle’s representatives after the end of the war.^120^

Still, *l’Aurore* received financial support from the Vichy government led by Philippe Pétain during the occupation of France. It also maintained a close relationship with the French ambassador, Henry Cosme (1885–1952), who had arrived in China before the occupation of France but remained in office under the Vichy regime. Cosme tried to protect French colonial interests in Asia, particularly in Indochina. In private, he declared that he personally sided with de Gaulle, but in his official capacity, he took measures against those who organised for the Resistance or went to join de Gaulle in London.^121^ Cosme was very supportive of *l’Aurore*, which he visited as soon as he took office. In 1941, he created a prize in his own name for deserving students of the medical faculty.^122^

Chinese faculty members took a more prominent role in the affairs of the institution as the war reduced the number of French faculty members. This was reflected in a greater interest in Chinese medicine, which had existed at *l’Aurore* at least since the 1930s but gained momentum during the war.^123^ Articles on traditional Chinese medicine appeared in the faculty *Bulletin*, revealing some interest in exploring how laboratory medicine could transform traditional ways of healing.^124^ This was in keeping with laboratory experiments carried out on ‘Chinese herbal drugs […] [i]n a process now known as ‘bioprospecting,’ [where] many drugs were analyzed for active principles that, once isolated, could be repackaged and sold as modern (i.e., Western-medical) drugs.’^125^ Anne Frédérique Glaise notes that very practical reasons also made traditional Chinese medicine more attractive to Aurorians in wartime, ‘as medicines are scarce and expensive, attention turns to Chinese medicine in the hope that it will provide alternatives to these shortages’.^126^ For ‘fixers’ in Shanghai, such as Xu Guanqun (1899–1972), producing and selling what they marketed as Chinese medicines approved by professional scientists became particularly lucrative.^127^

Using their knowledge of the local environment and a vast pharmacopoeia, Chinese members of the medical faculty could develop treatments and make public health measures more palatable to local people.^128^ For example, Liu Yongchun, an early Aurorian doctor and vaccination authority in Shanghai, studied the synthesis of acupuncture with neurology and vaccination. Regardless of the scientific validity of these methods, patients accepted vaccination more readily when injected at acupuncture points.^129^ In 1942 and 1943, the *Musée Heude* (the Museum of Natural History of *l’Aurore*) opened an exhibition on Chinese materia medica, the impact of which was amplified by publications and lectures.^130^ These tentative explorations of traditional Chinese medicine in some ways foreshadowed programmes in the Maoist period that combined Western and Chinese medicine.^131^

In 1943, *l’Aurore*’s medical faculty entered into an agreement that allowed a Society for the Study of Chinese Medicine (*Société de recherche sur la médecine chinoise*) to use its laboratories.^132^ This agreement was likely facilitated by the development of bacteriology around the new Pasteur Institute founded in Shanghai in 1938.^133^ *L’Aurore* had always been close to the Pasteurians, not least because of the training of some of its professors, and had maintained links with the Pasteur Institutes in Indochina.^134^ Inaugurated during a cholera outbreak, the Pasteur Institute in the French Concession of Shanghai became a vital public health facility, particularly for vaccination.^135^

It was therefore through wartime, during the last fifteen years of its existence, that the medical faculty reached its highest stage of development. Immediately after the war, Aurorians established friendly contact with doctors arriving aboard American and British military ships.^136^ The university benefited from post-war investment in reconstruction, but not necessarily from French sources as before. The French Concession in Shanghai had ceased to exist, and funding from the French government dwindled, but the Jesuits’ property holdings continued to provide a financial base for *l’Aurore*.^137^ From this position of relative strength, *l’Aurore* continued to exist for seven years after the war, as China plunged into a civil war between the Nationalists led by Chiang Kai-Shek and the Communists led by Mao Zedong. Some French professors arrived, and relations with France were partially restored, but more and more Aurorians were leaving the country.^138^ Shanghai fell to the Communists in late May 1949, and Mao proclaimed the People’s Republic of China in October of the same year. Soon after the Communists took Shanghai, *l’Aurore* came under financial and political pressure. The Communist view, which had become dominant in Chinese national politics, was that *l’Aurore* was a demonstration of Western cultural imperialism in China. A growing number of Aurorians were siding with the Communist regime.^139^ Ideological and financial pressure intensified with China’s entry into the Korean War in October 1950.^140^

As a result, in 1952, the faculty was dissolved and merged with St. John’s University and Tong De Medical College to form a new Chinese institution in line with the ruling regime’s priorities, the Shanghai Second Medical College – today the Shanghai Jiao Tong University School of Medicine.^141^ By contrast, Fudan University, also founded by Ma Xiangbo, continues to operate separately to this day. Through the actions of Communist Party-affiliated groups, *l’Aurore*’s associated hospitals and clinics were brought under the control of the emerging bureaucracies of the Shanghai People’s Government. In the 1950s, there was an attempt to import a Soviet model of medical education, but this was largely abandoned after thousands of ‘Soviet specialists left China in 1960’ according to Xi Gao.^142^ At that point, medical education throughout China had become more militarised and large scale. Its content reflected the Communist government’s policy of promoting Chinese medicine, leading to the opening of at least thirteen medical schools between 1955 and 1960 that explicitly taught traditional medicine.^143^

Because of their expertise, most Aurorian medical, nursing, and dental graduates who had not fled to Hong Kong or Taiwan continued to work in the new People’s Republic, although they were monitored for suspected political disloyalty and had to attend ideological indoctrination sessions. Like other former missionary institutions, *l’Aurore* provided specialised training and services that the state could not fully replace.^144^ In 1985, a group of Chinese alumni living in Shanghai founded an association of former Aurorians (*Amicale des anciens Auroriens*), based at the Second Medical University. Six of the eleven members of the association’s board worked in the medical sector in China.^145^

The lasting impact of *l’Aurore*’s medical faculty was thus based on its ability to make itself indispensable to public health in a context of constant warfare in China, and on the work of its Chinese members in its administration and medical activities. The medical faculty weathered the years of the Second Sino-Japanese War by officially concentrating on the medical aid it could provide and trying to adapt to the regime changes in China and France. This enabled it to reach a peak in recruitment and build new infrastructure. The war reduced French involvement in the faculty, allowing Chinese Aurorians to take on more responsibilities at *l’Aurore* and devote more attention to Chinese medicine using laboratory methods. After the war ended and the Communists came to power, *l’Aurore* continued to operate until it was dissolved by the government in 1952. Even then, many Aurorians continued to work in the Chinese medical profession because of their expertise, despite the fact that their background seemed incompatible with the policies of the Communist regime. The memory of the university was preserved largely through the initiative of doctors, including in an association.

## Conclusion

*L’Aurore* University in Shanghai was founded by the Jesuits in 1903, and it opened a medical programme in 1912. Over the next forty years, *l’Aurore*’s medical faculty trained hundreds of students, most of them Chinese. This article has argued that the durability and influence of *l’Aurore*’s medical faculty was due to its ability to rely on Jesuit missionaries in China without presenting itself as a missionary enterprise and to benefit from the support of the French imperial state without presenting itself as a formal imperial institution.

Although it was inseparable from the missionary work of the Jesuits, *l’Aurore* was only used as an instrument of explicit Catholic proselytism in one aspect: the medical ethics courses, which were rooted in Catholic principles and opposed birth control in particular. *L’Aurore*’s medical faculty also served the imperial ambitions of the French state, which at the time was particularly hostile to the clergy. The French authorities subsidised it to train students, especially Chinese, in French medicine in order to compete in medical education with the other imperial or private foreign powers active in China. The medical faculty consolidated its influence during the most perilous period of its existence: the years of war between China and Japan. Against a background of massive population movements, fighting, inflation, hunger, and the loss of staff to the military, the faculty increased its student numbers and built new infrastructure. Partly due to the more prominent role that the Chinese staff began to play during these years, teaching and research in Chinese medicine took on an unprecedented importance in the faculty. The Aurorians who remained in China after the Communist regime took over continued to be involved in public health, even founding an association in 1985 to bring together alumni and preserve *l’Aurore*’s legacy. Although not the most important foreign medical training institution in China at the time, *l’Aurore*’s faculty of medicine is therefore an odd case illustrating the complexity of the relationship between medical education and empire.

## Footnotes

1 We would like to thank the participants in the workshop *Teaching to Treat: Medical Education in the Empires, 18th-20th centuries* (University of Oxford, 21-22 June, 2021) that made this article possible, especially Wayne Soon, who acted as discussant. We are also grateful to the journal’s anonymous reviewers for their thoughtful comments, which greatly improved the analysis. Because the university analysed here used French as its main language, the French name of the university (*l’Aurore*) is used in this article. The preparation of this article was partly made possible by a postdoctoral contract from the *Institut francilien recherche, innovation, société* (IFRIS).

2 Yingjia Zhou, ‘L’Université l’Aurore (1903-1952), une création catholique française à Shanghai’, (unpublished master’s thesis : Université Lumière-Lyon 2, 2003), 26–7.

3 If the preparatory courses offered by *l’Aurore* are taken into account, this number must be approximately tripled.

4 The percentages are calculated using the total number of students in all faculties of the university for the years 1938–1945, without taking into account the preparatory courses offered at *l’Aurore.* This figure excludes the nursing programme. See: Archives jésuites, Vanves (henceforth AJ), *Diaire de l’Aurore, 1908–1948*, FCh322, 13 February and 20 September 1938, 6 March, 8 September and 24 October 1939, 3 March and 21 November 1940, 3 May, 9 September and 26 October 1941, 31 March 1942, 31 March 1943, 11 November 1943, 20 March and 5 November 1944, 30 April 1945. On the admission of female students, see: 27 November 1937. See also: AJ, FCh323, ‘Étudiants à l’Aurore, 1903–1951’.

5
*Aurora University, Shanghai. General Informations and Curriculum* (Shanghai: T’ou-Sè-Wè, 1935), 137 (the pagination used is the one at the bottom of the pages, not the one at the top).

6

Yi Ren and Mingzhe Zhu , ‘Finding God in All Things: Indirect Evangelization and Acculturation of Université l’Aurore in Modern China’, Religions, 14, 2 (2023), 199 . The ‘dual identity’ of medical education at *l’Aurore* is examined in Ren Yi, ‘The medical education of Shanghai Aurora University under the university’s interwoven dual French and Catholic identities’ [任轶, ‘法国和天主教双重身份交织下的上海震旦大学医学教育’ *Journal of the Social History of Medicine and Health*《医疗社会史研究》 2017 年第3期], 126–42.

7
*Ibid.*; Jean-Paul Wiest , ‘Bringing Christ to the Nations: Shifting Models of Mission Among Jesuits in China’, The Catholic Historical Review, 83, 4 (1997), 654–81.

8 Wiest, *op. cit.* (note 7), 669–674; Ren and Zhu, *op. cit.* (note 6), 9; Zhou, *op. cit.* (note 2), 29–30; Anne Frédérique Glaise, ‘L’évolution sanitaire et médicale de la Concession française de Shanghai entre 1850 et 1950’ (unpublished PhD thesis : Université Lumière Lyon 2, 2005), 367.

9
*Archives Nationales d’Outre-Mer* (henceforth ANOM), ‘French Colonial Empires’, http://www.archivesnationales.culture.gouv.fr/anom/en/Presentation/Empires-coloniaux-francais-11.html, accessed 8 March 2024.

10 For a recent historiographical overview on *l’Aurore*, see: Ren and Zhu, *op. cit.* (note 6), 2–3. The *Bulletin* corpus analysed here is incomplete but representative.

11 This paragraph draws on Steven Pieragastini , ‘A French University in China? The Forgotten History of Zhendan University (*Université Aurore*, 震旦大学 *Zhendan daxue*)’, Outre-Mers, Revue d’histoire, 105, 394–5 (2017), 90–2.

12 Ruth Hayhoe, ‘Towards the Forging of a Chinese University Ethos: Zhendan and Fudan, 1903-1919’, *The China Quarterly*, 94, 1983, 327–8. See also: AJ, *Diaire*, *op. cit.* (note 4), several dates, including notably 3 February 1933 and 18 March 1948.

13 Glaise, *op. cit.* (note 8), 367; Ren and Zhu, *op. cit.* (note 6), 4; Hayhoe, *op. cit.* (note 12), 324–5 and 329–31; Zhou, *op. cit.* (note 2), 24; Ren Yi , ‘L’éducation au service de la puissance chinoise: Ma Xiangbo et la création de l’Institut L’Aurore (1903–1905)’,Outre-Terre, 4, 45 (2015), 288–292 ; *Université l’Aurore, Shanghai. Renseignements généraux & organisation des études* (Shanghai: Imprimerie de T’ou-Sè-Wè,1934), 4.

14 Wiest, *op. cit.* (note 7), 670–1; Zhou, *op. cit.* (note 2), 25–6; *Université l’Aurore, op.cit* (note 13), 5–7.

15
*Bulletin médical de l’Université l’Aurore* (henceforth BMUA), 13, March 1936 (unpaginated advertisement for the university’s publications) and 17, April–June 1937 (opening page); *Université l’Aurore, op.cit* (note 13), 10.

16 Wiest, *op. cit.* (note 7), 666–7; Ren and Zhu, *op. cit.* (note 6), 3–4. See also: Mary Augusta Brazelton, *China in Global Health: Past and Present* (Cambridge: Cambridge University Press, 2023), 6.

17 AJ, *Diaire*, *op. cit.* (note 4), 8 July 1940, 30 June 1942, 8 July 1943, 11 July 1944, 7 July 1945; *Université l’Aurore, op.cit* (note 13), 27.

18 BMUA, 21, May–June 1938, 184; *Université l’Aurore, op.cit* (note 13), 91.

19 BMUA, 11, March 1935, 242–4; VI/5, September–October 1941, 305; Glaise, *op. cit.* (note 8), 274.

20
*Aurora University*, *op. cit.* (note 5), 124; *Université l’Aurore, op.cit* (note 13), map in the introductory pages.

21 Wiest, *op. cit.* (note 7); Ren and Zhu, *op. cit.* (note 6).

22 AJ, *Diaire*, *op. cit.* (note 4), 23 December 1947. See also: Wiest, *op. cit.* (note 7), 674.

23 AJ, *Diaire*, *op. cit.* (note 4), 8 July and 21 November 1940, 26 October 1941, 30 June and 22 October 1942, 8 July and 11 November 1943, 11 July and 5 November 1944, 7 July 1945, 25 June and 23 December 1947.

24
*Ibid.*, 16 October 1936, 25 December 1947; Glaise, *op. cit.* (note 8), 380.

25 For instance: BMUA, V/6, November–December 1940, 412; VI/2, March–April 1941, 134; X/4, July–August 1945, 240. See also: *Aurora University*, *op. cit.* (note 5), 114–5; Wang Weijia, ‘To Blaze a New Trail: an Outstanding University. Research on Shanghai Zhendan University (1903-1952)’, (unpublished PhD thesis: Central China Normal University Modern History Institute, 2003) [王薇佳, ‘独辟蹊径:上海震旦大学研究 (1903 – 1952)’], 81.

26 BMUA, VIII/3, May–June 1943, 228; X/1–2, January–April 1945, 104; AJ, *Diaire*, *op. cit.* (note 4), 30 September 1944.

27 ANOM, INDE 340 158; *Journal officiel de la République française*, 184, 8 July 1904, 4129–30 and 336, 11 December 1905, 7205–9.

28 BMUA, V/4, July–August 1940, 195–6; *Aurora University*, *op. cit.* (note 5), 132; Ren and Zhu, *op. cit.* (note 6), 8.

29

P.G. Payen , Déontologie médicale d’après le droit naturel, devoirs d’état et droits de tout médecin (Shanghai: Imprimerie de la Mission Catholique, 1922). The information on the different editions is taken from WorldCat. Payen’s work also inspired the Aurorian doctor Victor Smolnikof to write a book on the same subject in Russian: BMUA, IV/1, January–February 1939, 78–9.

30

Michelle T. King , ‘Margaret Sanger in Translation: Gender, Class, and Birth Control in 1920s China’, Journal of Women’s History, 29, 3 (2017), 61 ; Sarah Mellors Rodriguez , Reproductive Realities in Modern China. Birth Control and Abortion, 1911–2021 (Cambridge: Cambridge University Press, 2023), 19 ; Mirela Davis , ‘“The Task is Hers:” Going Global, Margaret Sanger’s Visit to China in 1922’, Asia Pacific Perspectives, 14, 1 (2016), 75 .

31 Davis, *op. cit.* (note 30), 90.

32

Yuehtsen Juliette Chung , ‘Eugenics in China and Hong Kong: Nationalism and Colonialism, 1890s–1940s’, in Alison Bashford and Philippa Levine (eds), The Oxford Handbook of the History of Eugenics (Oxford: Oxford University Press, 2010), 258–73; Brazelton, *op. cit.* (note 16), 27.

33 Anne Carol, *Histoire de l’eugénisme en France. Les médecins et la procréation, XIX^e^-XX^e^ siècle* (Paris: Seuil, 1995), 77–84.

34 King, *op. cit.* (note 30), 61.

35 AJ, *Diaire*, *op. cit.* (note 4), 28 February 1936; King, *op. cit.* (note 30), 78; Rodriguez, *op. cit.* (note 30), 25.

36 BMUA, 13, March 1936, foreword.

37 BMUA, 9, February 1934, 61–80 and 11 (supplement), February 1935.

38 All references to Payen’s book in this paragraph are taken from: BMUA, 9, February 1934, 61–80.

39 BMUA, 9, February 1934, 69 – Martin Robert’s translation from French.

40 Payen, *op. cit.* (note 29), 427–9.

41
*Aurora University*, *op. cit.* (note 5), 133.

42 Ling Ma, ‘Gender, Law, and Society: Abortion in Early-Twentieth-Century China’ (unpublished PhD Dissertation: State University of New York – Buffalo, 2016), 132.

43
*Shanghai Health Gazetteer*, Part 18 – Personnages, Chapter 2 – Directory, Section 2 – Western Medicine [上海卫生志 第十八篇人物 第二章名录 第二节 西医]; https://www.shtong.gov.cn/difangzhi-front/book/detailNew?oneId=1&bookId=67643&parentNodeId=67774&nodeId=64998&type=-1

44 Li Qiang, ‘Medical education in missionary universities and the construction of medical ethics in modern China: A preliminary study on the relevant works of Yu Fengbin and Song Guobin’; [李强, ‘教会大学医学教育与近代中国医学伦理建设——对俞凤宾、宋国宾相关论著的初探’] *Journal for Research of Christianity in China* [中国基督教研究], 9 (2017), 194–209; D. E. Mungello, *Drowning Girls in China: Female Infanticide in China since 1650* (Lanham: Rowman & Littlefield Publishers, 2008); Henrietta Harrison , ‘“A Penny for the Little Chinese”: The French Holy Childhood Association in China, 1843–1951’, American Historical Review, 113, 1 (2008), 76–8.

45 Ma, *op. cit.* (note 42), 129–33.

46 Florence Bretelle-Establet, ‘Resistance and Receptivity: French Colonial Medicine in Southwest China, 1898-1930’, *Modern China*, 25, 2 (April 1999), 171–203; François Joyaux, *Nouvelle histoire de l’Indochine française* (Paris: Perrin, 2022), 143.

47 Florence Bretelle-Establet, *La santé en Chine du Sud (1898-1928)* (Paris: CNRS Éditions, 2002), 20–2; Bridie Andrews , The Making of Modern Chinese Medicine, 1850-1960 (Vancouver: UBC Press, 2014), 19 .

48 Joyaux, *op. cit.* (note 46), 143–61; Chien-Ling Liu, ‘Relocating Pastorian Medicine: Accommodation and Acclimatization of Medical Practices at the Pasteur Institutes in China, 1899–1951’ (unpublished PhD thesis: University of California, Los Angeles, 2016), 102–4.

49 Sarah Legrandjacques, ‘L’enseignement supérieur en Asie française et britannique: expériences croisées à l’âge des Empires (Années 1850-1930)’, *Outres-Mers*, 1, 394–5, (2017), 46–52. The relevant sources are the following at the ANOM: GGI 6779, GGI 11390, GGI 6780. See also: Brazelton, *op. cit.* (note 16), 8.

50 ANOM GGI 6779 – Martin Robert’s translation from French. See also: ANOM GGI 51054.

51

Laurence Monnais-Rousselot , Médecine et colonisation. L’aventure indochinoise, 1860–1939 (Paris: CNRS Éditions, 1999), Chap. VII.

52 ANOM GGI 11390; *Bulletin des actes administratifs des Établissements français dans l’Inde*, 36/30 (Pondichéry: Imprimerie du Gouvernement, 1863), 91–5. See also: Legrandjacques, *op. cit.* (note 49), 44–6.

53 ANOM GGI 6779.

54 All the following at the ANOM: GGI 20299, GGI 23742, GGI 51046.

55 Bretelle-Establet, *op.cit.* (note 47), 25, 147, and 157–60; Florence Bretelle-Establet , ‘Diplomatie et politique coloniale. La médecine française au Yunnan de 1898 à 1931 d’après les sources coloniales françaises et des études chinoises’, Outre-Mers. Revue d’histoire, 315 (1997), 41 . See also: Kim Girouard, ‘Faire l’histoire de la médicalisation en Chine du Sud au début du XX^e^ siècle: l’expérience des sources’, *Cahiers d’histoire*, 37, 2 (2020), 43–64.

56
*Archives diplomatiques de Nantes*, 691PO/1/114–8. See also: Dominique Richard-Lenoble , Martin Danis , and Pierre Saliou , ‘La médecine tropicale d’hier à aujourd’hui’, Bulletin de l’Académie nationale de médecine, 197, 7 (2013), 1358 25796727.

57 Jean Louis Bussière, ‘Jean Augustin Bussière (1872–1958). Un trait d’union entre la France et la Chine’, *Histoire des sciences médicales*, LII, 1 (2018), 100–1; *Journal officiel des établissements français dans l’Inde*, 51/43, 26 October 1900 (kept at ANOM INDE 550 1101); ‘Biographie’, *Association Jean-Augustin Bussière*, https://jeanaugustinbussiere.wordpress.com/bio/, accessed 23 March 2024.

58 ANOM GGI 54978.

59 Bussière, *op. cit.* (note 57), 102–5; BMUA, XIII/1, January–March 1948, 78.

60 Ren and Zhu, *op. cit.* (note 6), 6.

61
*World Directory of Medical Schools*, https://www.wdoms.org/, accessed 23 March, 2024; ‘Dr. Erich Paulun’, China Center Tübingen: https://t.ly/J3tyb and ‘History’, HKU Med: https://t.ly/6YVMj, both accessed 23 March 2024; Kim Girouard , ‘Opening Doors for Men: Women’s Medical Education in South China, 1899–1936’, in Delia Gavrus and Susan Lamb (eds), Transforming Medical Education. Historical Case Studies of Teaching, Learning, and Belonging in Medicine (Montreal-Kingston: McGill-Queen’s University Press, 2022), 281–304 ; TW Wong , ‘Dr Man-kai Wan and the Medical Students of the Hong Kong College of Medicine at Nethersole Hospital in 1898’, Hong Kong Medical Journal, 27, 6, (2021), 266–834949738 10.12809/hkmj-hkmms202112; Faith CS Ho , ‘The Beginning of Medical Education in Hong Kong 125 years ago… Its Unique Features in Comparison with Similar Efforts in China’, Hong Kong Medical Journal, 18, 6 (2012), 544–5023223661 ; Wiest, *op. cit.* (note 7), 677; John Z. Bowers , ‘The Founding of Peking Union Medical College: Policies and Personalities’, Bulletin of the History of Medicine, XLV, 4 (1971), 305–94937636; *The China Medical Journal*, XL, 8 (August 1926), 699–799. See also: Xi Gao , ‘Foreign Models of Medicine in Twentieth-Century China’, in Bridie Andrews and Mary Brown Bullock (eds), Medical Transitions in Twentieth-Century China (Bloomington: Indiana University Press, 2014), 185–90; Legrandjacques, *op. cit.* (note 49), 51.

62

Pierre Huard , ‘Medical Education in South-East Asia (Excluding Japan)’, in C. D. O’Malley (ed.), The History of Medical Education (Los Angeles: University of California Press, 1970), 373 .

63
*Archives diplomatiques de Nantes*, 635/po/b72, cited in ‘Enjeux propagandistes de l’Université l’Aurore à Shanghai,’ *L’atelier numérique de l’histoire*, ENS Lyon, https://shorturl.at/pzEY7, accessed 10 July 2023.

64 H. Balme, ‘The history of Western Medical Education in China’, *The China Medical Journal*, XL, 8, (1926), 703; Lincoln C. Chen, Michael R. Reich, and Jennifer Ryan, ‘History and development of medical education in East Asia: An overview’, in Lincoln C. Chen , Michael R. Reich , and Jennifer Ryan (eds), Medical Education in East Asia: Past and Future, (Bloomington: Indiana University Press, 2017), 3–19 ; *China Medical Board*, ‘Who We Are’, https://chinamedicalboard.org/who_we_are, accessed 25 March 2024. See also: Brazelton, *op. cit.* (note 16), 16–7.

65 AJ, *Diaire*, *op. cit.* (note 4), 20 February, 20 and 22 March, 6 and 9 May, and 15 June 1933; 25 March and 20 December 1934; 23–5 January, 9–15 February and 19 March 1935. See also: Glaise, *op. cit.* (note 8), 332–3.

66 For example: BMUA, 15, October 1936, index to advertisers.

67 Gao, *op. cit.* (note 61), 174–7; Andrews, *op. cit.* (note 47), 149–66.

68 Andrews, *op. cit.* (note 47), 164–71; Brazelton, *op. cit.* (note 16), 25–7.

69 Andrews, *op. cit.* (note 47), 20–1. See also: Chieko Nakajima , Body, Society , and Nation . The Creation of Public Health and Urban Culture in Shanghai (Cambridge, Massachusetts: Harvard University Asia Center, 2018), 17 .

70 Glaise, *op. cit.* (note 8), 366; Zhou, *op. cit.* (note 2), 32; AJ, FCh324.

71 Wiest, *op. cit.* (note 7), 672–8; Glaise, *op. cit.* (note 8), 369; AJ, FCh323, ‘Une université française en Chine’; AJ, FCh324.

72 BMUA, 5, March 1931, ‘Le docteur Sibiril’; Glaise, *op. cit.* (note 8), 370–1; Gao, *op. cit.* (note 61), 190–4.

73 BMUA, 21, May–June 1938, 185–6.

74 BMUA, VI/5, September–October 1941, 286 and 341.

75 BMUA, IX/6, November–December 1944, 460; *Aurora University*, *op. cit.* (note 5), 108.

76 Glaise, *op. cit.* (note 8), 373–4; BMUA, 21, May–June 1938, 184.

77 BMUA, 18, July–December 1937, 120–34.

78 Ren and Zhu, *op. cit.* (note 6), 10; Glaise, *op. cit.* (note 8), 369; *Université l’Aurore, op.cit* (note 13), 25; Hayhoe, *op. cit.* (note 12), 333.

79 BMUA, 21, May–June 1938, 185–7. See also: Zhou, *op. cit.* (note 2), 32–4; Glaise, *op. cit.* (note 8), 374; Ren and Zhu, *op. cit.* (note 6), 6.

80

David Luesink , ‘Anatomy and the Reconfiguration of Life and Death in Republican China’, Journal of Asian Studies, 76, 4 (2017), 1019–20 (cited in Clément Fabre, ‘Bruits de Chine. Intolérances auditives occidentales dans la nuit chinoise du XIX^e^ siècle’, *Socio-Anthropologie*, 41 (2020), footnote 21); Gao, *op. cit.* (note 61), 180.

81
*Aurora University*, *op. cit.* (note 5), 127.

82 AJ, *Diaire*, *op. cit.* (note 4), 2 October 1934; 15 March 1941; 15, 16, and 21 January, 15 February, and 20 September 1943. See also: AJ, FCh324, 10 October 1916.

83

Christian Henriot , ‘“Invisible deaths, silent deaths”: “Bodies without masters” in Republican Shanghai’, Journal of Social History, 43, 2 (2009), 413 . See also: Nakajima, *op. cit.* (note 69), 98.

84

Wayne Soon , Global Medicine in China. A Diasporic History (Stanford: Stanford University Press, 2020), 179–80.

85 BMUA, 9, February 1934, 101; Glaise, *op. cit.* (note 8), 374–5; *Aurora University*, *op. cit.* (note 5), 138–42.

86 ‘The Nursing School of Zhendan University’, *Catholic Women*, 5, 2 (1938), 162; *Shanghai Gazetteer of Religion*, Part 4-Catholicism, Chapter 3-Education, Section 4-General Education.

87 AJ, *Diaire*, *op. cit.* (note 4), 26 October 1941. See also: BMUA, XI/2–3, March–June 1946, 179.

88 All the following examples are from the BMUA: X/1–2, January–April 1945, 104; XIII/2, April–June 1948, 163.

89 Zhou, *op. cit.* (note 2), 26; AJ, FCh323, ‘Note sur la Faculté de médecine de l’Université l’Aurore’; all the following in the BMUA: 15, October 1936, 12; X/4, July–August 1945, 237; VI/5, September–October 1941, 357. The links with Lyon are perhaps not unrelated to the fact that there was a Franco-Chinese Institute there (1921–1950): ‘L’histoire de l’IFCL’, *Bibliothèque municipale de Lyon*, https://t.ly/ruV-G; ‘L’Institut franco-chinois de Lyon (IFCL)’, *Patrimoine Lyon*, https://t.ly/BlU1g; ‘Institut franco-chinois, 326J 1-326J142’, *Archives du département du Rhône et de la métropole de Lyon*, https://t.ly/Y3H2U, all accessed 25 March 2024.

90 BMUA, 9, February 1934, 93–7.

91 BMUA, X/1–2, January–April 1945, 103.

92 AJ, *Diaire*, *op. cit.* (note 4), 31 March 1942.

93 Glaise, *op. cit.* (note 8), 245–64; BMUA, V/5, September–October 1940, 328 and IX/6, November–December 1944, 459–60.

94 Glaise, *op. cit.* (note 8), 374; all the following in the BMUA: 9, February 1934, 90–2; IV/2, March–April 1939, 177; V/3, May–June 1940, 192; VII/1, 1942, 328; X/5–6, September–December 1945, 367; XI/4–5–6, July–December 1946, 280; XII/5–6, November–December 1947, 308.

95
*Bulletin de l’Université l’Aurore*, 16, *Supplément médical*, October 1928; all the following in the BMUA: 11, March 1935, 237–41; 23, September–October 1938, 338; 24, November–December 1938, 415–6; IV/2, March–April 1939, 147; IV/3, May–June 1939, 267–8; VI/4, July–August 1941, 280–1; VI/5, September–October 1941, 355; VIII/1, January–February 1943, 74; VIII/6, November–December 1943, 460 and 473; IX/6, November–December 1944, 445 and 458.

96
*Aurora University*, *op. cit.* (note 5), 61; all the following in the BMUA: IV/2, March–April 1939, 177; V/5, September–October 1940, 345; VI/6, November–December 1941, 427; VIII/1, January–February 1943, 73 and 76; VIII/2, March–April 1943, 150; VIII/3, May–June 1943, 229; IX/6, November–December 1944, 457 and 463; X/4, July–August 1945, 238–9.

97 BMUA, VI/5, September–October 1941, 353.

98 All the following in the BMUA: XII/5–6, November–December 1947, 306; XIII/1, January–March 1948, 75; XIII/2, April–June 1948, 160; XIII/2, April–June 1948, 159–60.

99 Glaise, *op. cit.* (note 8), 368; Hayhoe, *op. cit.* (note 12), 333–4.

100 Zhou, *op. cit.* (note 2), 22; Ren and Zhu, *op. cit.* (note 6), 10.

101 Glaise, *op. cit.* (note 8); Nakajima, *op. cit.* (note 69), 29–31; Liu, *op. cit.* (note 48), Chapter III. See also: Kerrie L. Macpherson , A Wilderness of Marshes: The Origins of Public Health in Shanghai, 1843–1893 (New York: Lexington Books, 2002); Xincheng Shen , ‘Engineering Shanghai: Water, Sewage, and the Making of Hydraulic Modernity’ (unpublished PhD thesis: Georgia Institute of Technology, 2019); Ruth Rogaski , Hygienic Modernity: Meanings of Health and Disease in Treaty-Port China (Berkeley: University of California Press, 2004).

102 Glaise, *op. cit.* (note 8), 348–54 and 370; Zhou, *op. cit.* (note 2), 20; *Université l’Aurore, op.cit* (note 13), 15 and 18.

103 Hayhoe, *op. cit.* (note 12), 334–5; Wiest, *op. cit.* (note 7), 674–6. See also: Zhou, *op. cit.* (note 2), 23; Yang Zhen, ‘Relations entre intellectuels et pouvoir politique à travers le *Bulletin de l’université l’Aurore* (1916–1929). À partir d’une image de François Villon construite par la revue’, in Michel Espagne and Li Hongtu (eds), *Chine France – Europe Asie. Itinéraire de concepts* (Paris: Éditions Rue d’Ulm, 2018), 534.

104
*Aurora University*, *op. cit.* (note 5), 110.

105
*Relations de Chine*, 30, 4 (October 1932), 35–43.

106 Ren and Zhu, *op. cit.* (note 6), 9.

107
*Annual Report of Shanghai International Relief Society [1937*–*38], Compilation of Historical Materials on China’s National Conditions during the War of Resistance Against Japanese Aggression*, 10 (National Library of China Document Microfilm Copy Center, 2006) [抗日战争时期中国国情史料汇编].

108 Ren Yi, ‘A Brief Analysis of the Rescue of Refugees by the French Concession Authorities and the Catholic Church during the Two Anti-Japanese Conflicts of the 1930s’, *Republican Archives* 119 (2015), 63–71 [任轶, 浅析两次淞沪抗战时期法租界当局与天主教会对难民的救助, 民国档案]; all the following in the BMUA, IV/1, January–February 1939, 73; VI/2, March–April 1941, 128; VIII/1, January–February 1943, 73.

109 Dr Palud and Dr Ge Jing, ‘A Survey of Health Work in the Shanghai French Concession in 1938 (Chinese and French Bilingual), *Zhendan Yikan*, 4, 2 (1939), 133–46; BMUA, 24, November–December 1938, 365–74; Brazelton, *op. cit.* (note 16), 30–2.

110 AJ, *Diaire*, *op. cit.* (note 4), 15 November 1937; 29 February and 14 December 1944.

111 Glaise, *op. cit.* (note 8), 376.

112 BMUA, X/1–2, January–April 1945, 101.

113 On the school of dentistry: BMUA, V/1, January–February 1940, 69–70.

114 All the following in the BMUA: IV/5, September–October 1939, 429; V/3, May–June 1940, 188; V/5, September–October 1940, 343; VI/4, July–August 1941, 277; VI/5, September–October 1941, 314 and 354; VIII/3, May–June 1943, 227.

115 Glaise, *op. cit.* (note 8), 362–5; BMUA, IV/6, November–December 1939, 516 and V/3, May–June 1940, 191–2.

116

Nicole Bensacq-Tixier , La France en Chine de Sun Yat-Sen à Mao Zedong, 1918–1953 (Rennes: Presses Universitaires de Rennes, 2014), Chapters IV–VII; Nicole Bensacq-Tixier, *Dictionnaire biographique des diplomates et consuls en Chine* (Rennes: Presses Universitaires de Rennes, 2013), 119–24. For the number of French people in Shanghai in 1942, see: Christine Cornet, ‘The bumpy end of the French concession and French influence in Shanghai, 1937–1946’, in Christian Henriot and Wen-Hsin Yeh (eds), *In the Shadow of the Rising Sun. Shanghai under Japanese Occupation* (Cambridge: Cambridge University Press, 2004), 261.

117 AJ, *Diaire*, *op. cit.* (note 4), 12–3 July 1940; 24 August 1944; all the following in the BMUA: V/1, January–February 1940, 69–70; V/2, March–April 1940, 130; XI/1, January–February 1946, 1–10; XII/3–4, August–October 1947, 224.

118
*Archives Nationales de France*, BB/27/1436, https://www.siv.archives-nationales.culture.gouv.fr/siv/UD/FRAN_IR_057228/denat8493, accessed 22 March, 2024.

119 BMUA, VI/5, September–October 1941, 357 and X/4, July–August 1945, 237; AJ, *Diaire*, *op. cit.* (note 4), 4 August 1941.

120
*Le Devoir* (Canada), 23 October 2006, B2. Dr Chabaud, Director of Shanghai’s Pasteur Institute and professor of microbiology at *l’Aurore*, was made a *Chevalier* of the Legion of Honour for his wartime involvement in the underground Resistance in Paris: BMUA, XII/2, April–July 1947, 144.

121 Bensacq-Tixier, *op. cit.* (note 116, both references).

122 AJ, *Diaire*, *op. cit.* (note 4), 9 April 1939; all the following in the BMUA: IV/3, May–June 1939, 267; VI/4, July–August 1941, 277; VIII/3, May–June 1943, 227.

123
*Aurora University*, *op. cit.* (note 5), 116–7; all the following in the BMUA: 7, October 1932, 1–56; 8, June 1933, 57–88; 11, March 1935, 89–112; 12, August 1935, 113–128; 14, June 1936, introduction page; IV/3, May–June 1939, introduction page

124 All the following in the BMUA: IV/4, July–August 1939, 317–9; V/1, January–February 1940, 58–60; VII/1, 1942, 1–14; IX/5, September–October 1944, 334–42. See also: AJ, *Diaire*, *op. cit.* (note 4), 24 October 1934.

125 Andrews, *op.cit.* (note 47), 58, see also 87 and 212. On the history of Chinese medicines, particularly in the late imperial period, see the work of Martha Hanson.

126 Glaise, *op. cit.* (note 8), 364 – Martin Robert’s translation from French.

127 Sherman Cochran, ‘Marketing medicine across enemy lines. Chinese “fixers” and Shanghai’s wartime centrality’, in Henriot and Yeh (eds), *op. cit.* (note 118), 66–89.

128 This is the argument of Liu, *op. cit.* (note 48), which finds echoes in the history of *l’Aurore*’s medical faculty.

129
*Ibid.*, 63–79, 97–9, 214.

130 Glaise, *op. cit.* (note 8), 362; BMUA, VIII/1, January–February 1943, 74; AJ, *Diaire*, *op. cit.* (note 4), 26 February 1943. The *Musée Heude* was founded in the mid-nineteenth century and incorporated into the *l’Aurore* in 1930: Ren and Zhu, *op. cit.* (note 6), 4.

131 On this topic, see for example: Xiaoping Fang , Barefoot Doctors and Western Medicine in China (Rochester, NY: University of Rochester Press, 2013).

132 AJ, *Diaire*, *op. cit.* (note 4), 31 July 1943.

133 BMUA, 20, March–April 1938, 117–21; Aro Velmet, *Pasteur’s Empire: Bacteriology and Politics in France, its Colonies, and the World* (Oxford: Oxford University Press, 2020), 112–3. See also: Liu, *op. cit.* (note 48), introduction and chapter I.

134 BMUA, 9, February 1934, 81–5 and 10, August 1934, 146–71.

135 BMUA, 24, November–December 1938, 418; Liu, *op. cit.* (note 51), 149–51.

136 BMUA, X/4, July–August 1945, 234–5.

137 BMUA, XIII/2, April–June 1948, 157 and XIII/3–4, September–December 1948, 230.

138 All the following in the BMUA: XI/1, January–February 1946, 68–71; XI/2–3, March–June 1946, 181–2; XII/3–4, August–October 1947, 227–9; XIII/1, January–March 1948, 76.

139 AJ, *Diaire*, *op. cit.* (note 4), 29 November 1945, 11 November 1947, 1 January 1948; AJ, FCh323, several typewritten or published documents.

140

Steven Pieragastini , ‘Reform and Closing Up: Thought Reform and the Institutional Reorganization of Shanghai’s Universities’, Twentieth-Century China, 43, 2 (2018), 139–62.

141 AJ, FCh323, letter dated 26 June 1953; ‘About Shanghai Jiao Tong University School of Medicine’, https://www.shsmu.edu.cn/english/About/About_SJTUSM.htm, accessed 23 March 2024.

142 Gao, *op. cit.* (note 61), 196–201.

143
*World Directory*, *op. cit.* (note 61); Kim Taylor, *Chinese Medicine in Early Communist China, 1945*–*63: A Medicine of Revolution* (London: RoutledgeCurzon, 2005); Wayne Soon, *op. cit.* (note 84), 2–3, 126–7, 201–3; Glaise, *op. cit.* (note 8), 365.

144 Shanghai Municipal Archives B242-1-784 ‘Shanghai Municipal People’s Government Committee, Shanghai Municipal Health Bureau, Reply regarding the treatment of nuns in the First People’s Hospital, the Second Labor Hospital and the Andang Hospital’ [‘上海市人委, 上海市卫生局关于处理第一人民医院, 第二劳工医院及安当医院三个单位内修女的待遇问题的批复’], 11 March 1955.

145 AJ, FCh323, letter dated 27 Decembre 1985 and ‘Statuts de l’Amicale des Anciens Auroriens’.

